# Gut Microbiota and Metabolic Health: The Potential Beneficial Effects of a Medium Chain Triglyceride Diet in Obese Individuals

**DOI:** 10.3390/nu8050281

**Published:** 2016-05-12

**Authors:** Sabri Ahmed Rial, Antony D. Karelis, Karl-F. Bergeron, Catherine Mounier

**Affiliations:** 1 BioMed Research Center, Biological Sciences Department, University of Quebec at Montreal, Montreal, QC H2X 1Y4, Canada; rial.ahmed_sabri@courrier.uqam.ca (S.A.R.); bergeron.karl-frederik@uqam.ca (K.-F.B.); 2 Department of Exercise Science, University of Quebec at Montreal, Montreal, QC H2X 1Y4, Canada; karelis.antony@uqam.ca

**Keywords:** obesity, metabolically unhealthy obese, metabolically healthy obese, metabolic syndrome, non-alcoholic fatty liver disease, gut microbiota, *Bacteroidetes*, *Firmicutes*, endotoxemia, lipopolysaccharide, medium chain triglycerides, medium chain fatty acids

## Abstract

Obesity and associated metabolic complications, such as non-alcoholic fatty liver disease (NAFLD) and type 2 diabetes (T2D), are in constant increase around the world. While most obese patients show several metabolic and biometric abnormalities and comorbidities, a subgroup of patients representing 3% to 57% of obese adults, depending on the diagnosis criteria, remains metabolically healthy. Among many other factors, the gut microbiota is now identified as a determining factor in the pathogenesis of metabolically unhealthy obese (MUHO) individuals and in obesity-related diseases such as endotoxemia, intestinal and systemic inflammation, as well as insulin resistance. Interestingly, recent studies suggest that an optimal healthy-like gut microbiota structure may contribute to the metabolically healthy obese (MHO) phenotype. Here, we describe how dietary medium chain triglycerides (MCT), previously found to promote lipid catabolism, energy expenditure and weight loss, can ameliorate metabolic health via their capacity to improve both intestinal ecosystem and permeability. MCT-enriched diets could therefore be used to manage metabolic diseases through modification of gut microbiota.

## 1. Introduction

Obesity has become an international public health problem with 2.1 billion people worldwide being overweight (body mass index (BMI) ≥ 25.0) and more than half a billion among them being obese (BMI ≥ 30.0) [1,2]. Obesity is now described as a pandemic with increased prevalence in both adult and child populations. Since the 1980s, the combined prevalence of obesity and overweight increased by 28% in adults and 47% in children [1,2]. Obesity is a multifactorial affection with broad etiology, and multiple comorbidities. Most of these comorbidities are thought to be the result of aberrant body fat distribution leading to the metabolic syndrome [3,4,5]. This pathology is associated with an elevated waist circumference, a progressive state of non-alcoholic fatty liver disease (NAFLD) [3,4], insulin resistance, type 2 diabetes (T2D), some types of cancers (especially in women), hypertension, cardiovascular diseases, reproductive abnormalities, dyslipidemias, psychological affections [6], and a severely reduced life expectancy [3,7,8]. Obesity is also considered a risk factor for several other diseases such as chronic respiratory diseases [9] and arthritis [3].

Some contributing factors for obesity progression are: unfavorable genetic determinants [10], lack of physical activity [11], socio-economic status [1], circadian cycle disturbance [12], sleep deprivation [13], hormonal dysregulation [14], persistent organic pollutants [15] and alteration of the gut microbiota [16,17]. However, the most powerful inducer of obesity and its associated adverse metabolic effects remains, by far, inappropriate food intake [18]. The modern prevalence of obesity and metabolic syndrome is likely due to the rise in consumption of energy-dense food, containing high amounts of fat and carbohydrates [18], especially in Western countries. In these countries, fats typically account for 33% to 42% of dietary energy intake, with a rich proportion of long chain saturated fat [18]. When the energy intake exceeds both the caloric needs of the body and its glycogen storage capacity, dietary carbohydrates and fats are first converted and stored as triglycerides (TG) in white adipose tissue (WAT) and, later on, in other tissues such as the liver [19,20]. Sustained and abusive accumulation of lipids in the liver induces NAFLD (highly concurrent with obesity) which may result in lipotoxicity, steatohepatitis, hepatocyte cell death, fibrosis and eventually liver cirrhosis as well as hepatocarcinoma [21].

We aim here to overview the underlying causes of obesity and its associated metabolic abnormalities while paying special attention to a unique subset of the obese population: the metabolically healthy but obese (MHO) individuals. We will highlight the potential involvement of the gut microbiome as one of several contributors to the MHO status. We will finally introduce the idea of a medium chain triglycerides (MCT)-based dietary intervention with the aim of improving the metabolic state of metabolically unhealthy obese (MUHO) patients, either through modification of their intestinal health or by directly influencing lipid metabolism.

## 2. Etiology of Obesity and Associated Metabolic Complications

The current paradigm posits that fat accumulation leading to obesity results from an imbalance between energy intake and energy expenditure. Post-World War II food processing and marketing practices (quickly available energy-dense foods) and reductions in physical activity are the two main factors often blamed for the contemporary prevalence of obesity. As such, most treatment options involve creation of a negative energy balance, achieved by consuming fewer calories than energy expended. Alternatively, pharmacological and surgical therapies also exist [22]. Nevertheless, complex interactions among heredity [23], epigenetic imprinting [24], lifestyle, feeding behavior [25], as well as environmental [26] and physiologic inflammatory factors [27,28] also contribute to the etiology of obesity.

### 2.1. Comorbidities Related to Obesity

Most obese individuals fall into the MUHO category and are at increased risk for several diseases. They suffer from a severely reduced life expectancy: a 5.8 years average decrease for men and 7 years for women [29]. The visceral adipose tissue (typically abundant in obese individuals) secretes pro-inflammatory cytokines [27,28] that are, in part, responsible for obesity-associated pathogenesis [27,28]. The resulting metabolic syndrome defines a group of metabolic risk factors linked to health problems associated with overweight and obesity such as elevated visceral adiposity, larger waist circumference (>102 cm in men and >88 cm in women), hyper-triglyceridemia (>150 mg/dL) [5], reduced HDL (high density lipoprotein) cholesterol levels (<40 mg/dL in men and <50 mg/dL in women), elevated blood pressure (systolic > 130 mmHg and/or diastolic > 85 mmHg) and high fasting glucose levels [30]. A patient with at least three of these risk factors is currently diagnosed with the metabolic syndrome.

High levels of glycemia, associated with insulin resistance, are a hallmark of prediabetes and T2D, common diseases occurring in obese individuals suffering from the metabolic syndrome [5]. According to the most recent criteria, prediabetes is defined by a fasting glucose ranging between 100 and 125 mg/dL or between 140 and 199 mg/dL 2 h *post-prandium*, while these two values often exceed 126 mg/dL and 200 mg/dL in T2D, respectively [5]. In addition, the latest medical updates indicate that 50%–90% of patients with TD2 have a BMI > 25.0 (the overweight threshold) while those with a BMI > 35 have a 20-fold higher risk of developing T2D than healthy lean patients [31].

Risk of cardiovascular diseases, including atherosclerosis, coronary artery disease, stroke and pulmonary embolism, is higher in obese patients [32]. Obesity is also associated with a large spectrum of liver abnormalities including NAFLD [33]. Several additional comorbid disease risks are associated with obesity such as cholelithias, pancreatic failure sleep apnea, gynecological abnormalities, osteoarthritis, psychiatric illness as well as some types of cancer (breast, endometrial, prostate and colon) [22].

### 2.2. Metabolically Healthy but Obese Individuals (MHO)

There is a large body of evidence suggesting that not all obese individuals develop such important metabolic complications [34]. A unique subtype of obese individuals has been identified as MHO [35]. Despite excessive levels of body fat mass, MHO patients present higher levels of insulin sensitivity, a normal circulating lipid profile, no pronounced hepatic steatosis nor associated inflammatory state, no hypertension, and a lower visceral, muscle and hepatic fat content compared to MUHO subjects [36]. Since there is no standard definition for the identification of the MHO phenotype, the true prevalence of MHO individuals in the obese population is currently unclear. Depending on the method and the cut-off point used for certain metabolic risk factors, the prevalence of MHO subjects may range from approximately 3% to 57% of obese adults [37,38].

The favorable metabolic profile of MHO individuals has been associated with a lower risk of developing T2D or cardiovascular disease (CVD), and having a lower mortality risk compared to MUHO individuals [39]. There is also evidence refuting the notion that MHO subjects are totally protected from metabolic diseases [40]. The fundamental mechanisms underlying the different metabolic profiles of MHO and MUHO individuals remain poorly understood [41]. However, Berezina *et al*. showed that among patients with abdominal obesity, MHO phenotype was associated with a G45G Adiponectin genotype, while the T45T genotype for Adiponectin increased metabolic disorders risks [42]. This observation is all the more interesting since Adiponectin is a potent modulator of insulin sensitivity [43]. Also, in the latter decade, a consensus has emerged, which make the gut microbial population (also called the gut microbiota) a key determinant in the host metabolic profile [17,44]. The gut microbiota should therefore be taken into consideration in dietary and clinical approaches, for both MUHO and MHO treatments.

## 3. The Gut Microbiota: A Determining Factor for Metabolic State

### 3.1. Gut Microbiota Dysbiosis in Obesity

Current estimations indicate that the human intestine is colonized by a large population of above 100 trillion microbial cells organized in several taxa and constituting the gut microbiota [45]. The total biomass of the gut microbiota exceeds 1kg and is mainly concentrated in the large intestine, where about 10^12^ bacteria per gram of colonic tissue are found [46]. Such an important quantity of “foreign” cells implies a tightly regulated acceptance by the immune system of the host [46]. The gut microbiota plays a symbiotic role, ensuring several metabolic functions essential to the host. It is involved in the degradation of polysaccharides and oligosaccharides into simple metabolites such as short chain fatty acids (SCFA). These SCFA were recently found to promote intestinal impermeability [47] as well as hepatic sugar and lipid anabolism [48,49,50]. The gut microbiota is also an indispensable source of essential vitamins B and K [51]. Gut microbiota composition and diversity are subjected to important variations between individuals but also within the same individual under the influence of several physiologic and environmental factors such as antibiotic consumption, lifestyle and diet [52].

The alteration of gut microbiota is closely linked to tissue inflammation and to a broad range of metabolic abnormalities, including obesity and insulin resistance [52,53]. First observations suggested a link between the gut microbiota and the metabolic state of the host [54,55]. Several studies have since confirmed a direct implication of gut microbiota in obesity progression [17].

Backhed and colleagues first demonstrated that germ-free mice were protected against high fat and high sugar diet-induced obesity, via enhanced expression of hepatic and muscular Pgc-1α (a PPAR coactivator) and activation of lipid catabolism via AMPK activity [56]. Subsequently, Cani and collaborators established a close link between gut microbiota-induced “metabolic endotoxemia” and obesity-related insulin resistance in mice. They established the paradigm of gut microbiota-mediated metabolic endotoxemia inducing chronic low-grade of inflammation in the host. In obese mice, a dramatic change in caecum-resident bacteria characterized by a decrease in low lipopolysaccharide (LPS)-expressing bacterial taxa (such as *Bifidobacteria*) and concomitant elevation in high LPS-expressing taxa (gram-negative bacteria) was observed. This lead to increased concentration of circulating pro-inflammatory LPS [55]. A prolonged high fat diet was also reported to induce physiologic high-grade inflammation, weight gain and T2D progression by an LPS-dependent gut microbiota-associated endotoxemia mechanism in mice [56]. In addition, it was shown that genetically obese *ob/ob* mice display a lower endotoxemia and caecal LPS content following the inactivation of CD14, a cell receptor involved in inflammation [55,57]. Concomitantly, in a randomly recruited human male cohort, a link has been observed between circulating LPS and food intake [58].

In mice fed a high-fat diet, and in genetically obese *ob*/*ob* mice, suppression of gut microbiota via administration of a broad range of antibiotics resulted in a significant decrease of LPS caecal content. Consequently, fat accumulation and body weight were diminished, endotoxemia and inflammation at both systemic- and tissue-specific levels were blunted, and insulin sensitivity was improved [57]. Intriguingly, administration of antibiotics to livestock trough water and food is typically used to promote cattle and swine growth and weight-gain [59,60]. The mechanisms underlying this opposite effect are not known yet support a link between enteric bacteria and host metabolism, suggesting variable responses between animal species [59,61,62].

A dysbiosis induced by obesogenic factors adversely enhances intestinal permeability by modulating the expression of epithelial junction genes *zo-1* and *occludin* [63,64]. An optimal gut microbiota helps maintain intestinal barrier impermeability via the production of SCFA, which serve as metabolic precursors for colonocytes in normal physiologic hypoxia conditions [47,48,49]. Colonocytes require the catabolism of SCFA to potentiate the Hypoxia-inducible factor 1-dependent expression of key genes involved in biosynthesis [47,48,49]. This may explain why, in metabolic diseases such as obesity, an inappropriate microbiota adversely impacts intestinal permeability, leading to infiltration of bacterial LPS from gut lumen into intestinal epithelia, bloodstream and tissue (such as the liver) contributing to metabolic endotoxemia [47,48,49,65]. In fact, administration of antibacterial agents targeting caecal aerobic and anaerobic bacteria with high efficiency (namely norfloxacin and ampicillin) to mice fed a high-fat diet and *ob*/*ob* mice significantly enhanced their global glucose tolerance, decreased their fasting glycaemia and circulating LPS levels, and improved the secretion of Adiponectin [66], known for its positive effects on insulin sensitivity [43]. The current model of obesity-associated endotoxemia posits that gut-derived LPS and free fatty acids activate M1 macrophages which, together with other immune cells, create a hepatic and physiologic inflammatory process sustaining the above-mentioned obesity-related comorbidities [67].

Gut microbiota is now considered as a key factor of metabolic health [44,64]. The relative abundance of some bacterial phyla within the gut microbiota are associated with specific metabolic states, and subjected to the influence of diets [54]. In fact, quantitative identification of bacteria using the 16S ribosomal RNA gene showed that obesity is associated to a notable decrease in the relative abundance of *Bacteroidetes vs*. *Firmicutes* (from a proportion of 40:60 in lean patients or non-obese mice down to a proportion of 20:80) [54,68,69]. Interestingly, this ratio is similarly decreased in *ob/ob* mice, in mice subjected to obesogenic and diabetogenic diets, as well as in obese human subjects [68,70,71]. Moreover, within these two superkingdoms, bacterial subpopulations are themselves submitted to specific changes reflecting metabolic health, diet and antiobesity medical interventions such as bariatric surgery-induced weight loss [72,73,74]. These changes are diverse and still not well established (see Delzenne and Cani for a detailed review [69]).

While these observations established a definitive link between gut microbiota disturbance and metabolic diseases progression, it is still unclear which one of the two events induces the other. This question was addressed by several microbiota transplantation experiments [44,75]. The transplantation of fecal microbiota from obese mice and obese patients into germ-free lean mice transformed the lean mice into obese mice, while the germ-free mice receiving microbiota from lean mice did not show any metabolic symptoms related to obesity or insulin resistance [54,75,76,77]. These findings revealed a microbiota-mediated transmissible effect of diets and metabolic status, demonstrating that gut microbiota is not simply a collateral unit modulated by metabolic diseases but an active and potent modulator of metabolism. The gut microbiota may therefore play a role in the determination of metabolic status.

### 3.2. Correlation of MHO Metabolic Status with Gut Microbiota Profile

To date, very few animal studies described a clear role for gut microbiota in the establishment of the MHO phenotype. Serino *et al*. showed that different cohorts of mice issued from the same C57BL6 genetic background became either diabetic or resistant to diabetes and metabolic disorders despite being fed the same obesogenic high-fat diet. Compared to diabetic mice, resistant mice showed higher insulin sensitivity, lower inflammation, improved intestinal impermeability (in the ileum, caecum and colon) and lower adipogenesis [78]. Moreover, the metabolic profile of resistant mice was similar to the metabolic profile observed in mice fed a high fat diet supplemented with gluco-oligosaccharidic fibers. Oligosaccharidic fibers are commonly used to prevent high fat diet-induced endotoxemia, dysbiosis and obesity [79]. Interestingly, the resistant mice harbored a distinctive gut microbiota. The gut microbiota of the diabetic mice, in comparison with resistant mice, was characterized by a 20% decrease in the abundance of *Firmicutes* to the benefit of *Bacteriodetes*, mainly resulting from a dramatic decrease of the *lachnospiraceae* bacterial family. Within the *Bacteroidetes* phylum, the family of S24-7 bacteria was specifically increased (3-fold). In addition, compared to the diabetic mice, the microbiota of resistant mice presented an important decrease of the *helicobacter* genus, while *actinobacteria* remained stable [78]. The authors suggested that the gut microbiota could represent a “signature of the metabolic phenotype independent of differences in host genetic background and diets”. This suggestion is consistent with the results of a recent study performed in the brown bear (*Ursus arctos*) [80]. The bear is a mammal well known to accumulate very large amounts of adipose fat during the summer; developing hyperlipidemia while remaining metabolically healthy and resistant to atherosclerosis [81]. Sommer and colleagues showed that, during summer, bears harbored a different gut microbiota composition than during winter. Summer microbiota was enriched in *Firmicutes, Actinobacteria* and, in a lower proportion, enriched in proteobacteria while being depleted in *Bacteroidetes* [80]. Moreover, the abundance of several other bacterial families was also seasonally modulated. Interestingly, the transplantation of summer or winter microbiota from bear intestines to germ-free mice revealed a microbiota-dependent transmissible seasonal metabolic status as mice receiving summer microbiota showed a significant elevation of adiposity and body weight gain but a better glucose tolerance and a lower circulating TG level, suggesting an improved cardiometabolic state [80].

Completely ignored previously, the fungal subdivision of the gut microbiota (or “mycobiota”) has recently been revealed as a determinant of human metabolic state. As for bacteria, its specific composition seems to distinguish MHO from MUHO [82]. For example, Rodriguez and collaborators showed that obese patients with relative abundance of *Eurotiomycetes* > 1% in their gut, showed improved fasting insulinemia, insulin resistance index and circulating LDL-cholesterol levels [82].

There is a definite correlation between MHO status and intestinal flora. Gut microbiota remodelling could therefore be considered as a strategy to ameliorate metabolic health. Hence, we believe that a switch from MUHO into MHO metabolic state may be possible following gut microbiota remodelling, and that dietary interventions (such as those using prebiotic or bioactive nutrients) may help in this process.

## 4. MHO and MUHO: From Classical Dietary Interventions to a MCT-Based One?

Obese individuals have a lower success rate of maintaining weight loss after one year compared to non-obese individuals [83]. This high risk of weight regain may lead to a pattern of weight cycling [83], and this may be associated with metabolic complications as well as CVD and mortality [84,85]. In addition, significant weight loss can increase the levels of persistent organic pollutants (POPs) in the bloodstream [86,87] and may offset the beneficial effects of weight loss. In support of this idea, a recent study showed that obese subjects with the highest POP blood levels after weight loss suffered from a delay in the improvement of lipid and liver toxicity markers [88]. Therefore, health professionals may want to favor a metabolically healthy state instead of focusing on weight loss.

### 4.1. Weight Management in MHO Subjects

Several studies have examined the effect of lifestyle interventions, including diet and/or exercise training in MHO individuals. These have led to contradictory findings. Some have shown that, compared to MUHO, the metabolic profile of MHO individuals after weight loss did not improve following weight loss [89,90,91] while other studies have reported an improvement of several metabolic risk factors [92,93,94,95]. For example, a 9-month intensive lifestyle intervention program that consisted of Mediterranean diet nutritional counselling and high intensity interval training was associated with a reduction of metabolic risk factors (e.g., blood pressure, fasting glucose level) [94]. Furthermore, weight loss following laparoscopic adjustable gastric banding in MHO patients was associated with an improvement of insulin sensitivity levels after 6 months [96]. However, a significant decrease in insulin sensitivity was observed after weight loss in a different cohort of MHO individuals [97,98]. Finally, MHO women may lose less weight than MUHO subjects after 3 months on a low fat diet [99]. Based on the current evidence, it appears difficult to prescribe an optimal lifestyle intervention program to both MUHO and MHO individuals.

### 4.2. The Metabolic Protective Potential of Medium Chain Triglycerides (MCTs)

#### 4.2.1. MCTs as Bioactive Lipids

One angle of attack against metabolic diseases is the modification of the quality of dietary lipids. Such a nutritional strategy may be based on the consumption of “bioactive lipids”, such as mono-unsaturated or poly-unsaturated lipids, phytosterols, and free or esterified medium chain lipids [100,101]. We will emphasize our review on the metabolic benefits of medium chain free fatty acids (MCFA) and medium chain triglycerides (MCT).

Dietary medium chain fatty acids (MCFA) range between 6 and 10 carbon-chain lengths, including hexanoate (C6:0), octanoate (C8:0) and decanoate (C10:0). Large amounts of MCFA are found, in the form of MCT, in coconut and palm kernel oils where they account for more than 50% of total lipids [102,103]. They are also found in smaller amounts in bovine milk, where they can reach 14%–15% of the total lipid mass depending on cow breeds, types of pasture and seasonal conditions [104]. MCT differ from long chain triglycerides (LCT) by several physicochemical characteristics such as their smaller molecular size. MCT are hydrolyzed both faster and more extensively during digestion. Most of the remaining non-hydrolyzed MCT are readily absorbed by intestinal cells [103]. In addition, MCFA show greater solubility in aqueous media while remaining capable of passive, non-rate-limiting diffusion across cell membranes as a result of their relative short chains [100,101,102,103,105,106]. MCFA show a low affinity for anabolic enzymes (such as diglyceride acyltransferase) therefore undergoing minimal re-esterification, a process necessary for *de novo* synthesis of TG. Once absorbed during digestion, most of MCFA and MCT are transported through the portal system directly to the liver with minimal mobilization of chylomicrons while long chain fatty acids (LCFA) are packed in chylomicrons prior to their shipment to the periphery mainly via the lymphatic system [100,101,102,103,105]. Finally, in the liver, MCFA are preferentially metabolized to generate energy [107]. In fact, MCFA are known to enter cells passively before crossing mitochondria membranes, independently of the availability of the rate-limiting carnitine palmitoyltransferase 1 (CPT-1) transport system, to fuel the β-oxidation and ATP-generating pathways [105,106], induce thermogenesis and reduce *de novo* lipogenesis [108].

Because of these properties, MCT have been used for decades to overcome many metabolic and digestive abnormalities such as pancreatic insufficiency, fat malabsorption, impaired lymphatic chylomicron transport, severe hyperchylomicronemia, and total parenteral nutrition. MCTs are also used in preterm infant formulas [106].

#### 4.2.2. MCT-Supplemented Diets Prevent Obesity

##### (i) MCT and MCFA Exert Antilipogenic Effects

Using chick embryo hepatocytes, we have previously demonstrated, that free non-esterified hexanoate and octanoate significantly decrease insulin and T3 (triiodothyronine)-induced fatty acid synthase (FAS) expression and activity [108]. Activity of FAS, a key enzyme of *de novo* lipogenesis, is positively regulated in the post-prandial state by several hormones (such as insulin, ghrelin and T3) and nutrients (dietary lipid and carbohydrate derivatives) [109,110], and is an important contributor to obesity and NAFLD [20,111]. We and others have demonstrated that MCFA inhibit binding of transactivating receptors on the T3 response elements of FAS and Acetyl-CoA carboxylase (ACC) promoters [108,112,113,114]. Using LO2 hepatocytes, Wang and collaborators showed that the induction of cellular steatosis by LCT (esterified oleate and palmitate) is reversed by addition of either octanoate or decanoate. MCFA treatment shifts cells from global lipid anabolism to lipid catabolism and downregulates main *de novo* lipogenesis-activating transcription factors (Liver-X-receptor alpha and Sterol regulatory element binding protein-1), the lipogenic enzymes (ACC and FAS) and enzymes involved in fatty acid-uptake (Cluster of differentiation-36 and Lipoprotein lipase) [115].

##### (ii) The Cardiometabolic Protective Effects of Dietary MCT

The propensity of MCFA to be catabolized rather than esterified, highlights their metabolically beneficial potential. Several years ago, it was shown in rats that replacement of dietary LCT by octanoate-based MCT led to a significant attenuation of alcoholic steatosis associated with a decrease in *de novo* TG synthesis an adiposity, as well as elevated lipid oxidation [116]. Therefore, MCT-supplemented diets constitute a promising tool against adipogenic and steatogenic diseases. Feeding healthy rats with MCT-containing diets *vs*. diets containing LCT greatly decreases fat deposition without affecting whole-body protein content and assimilation [117]. Consumption of MCT is safe in regards to toxicological considerations and has been sanctioned by the FDA (Food and Drug Administration) for over 20 years [106,118]. Interestingly, dietary MCT promote post-prandial energy production. A single dose of MCT ranging between 5 and 50 g or a weeklong diet containing 40% MCT-fat systematically leads to elevated post-prandial oxygen consumption and thermogenesis, increased total lipid oxidation, higher energy expenditure and diminished energy storage in comparison with LCT administered under identical conditions [101,119,120,121,122,123]. Importantly, the propensity of MCT/MCFA to be degraded by oxidation is also observed in obese individuals [120,124].

Long-term replacement of LCT with TG composed of esterified MCFA and LCFA induces a measurable body fat loss without any impact on energy intake and global metabolism [101,125,126]. These specific TG, produced from the transesterification of MCT and LCT (S-MLCT) are interesting compounds because of their higher smoking temperature making them more appropriate for cooking purposes than simple mixtures of MCT and LCT. S-MLCT display a “L-M-L”-type structure (TG with LCFA in sn-1 position, MCFA in sn-2 and LCFA in sn-3), are preferable substrates for pancreatic enzymes and are therefore highly digestible [101,125,126]. Feeding healthy young volunteers for 3 months with either S-MLCT or LCT-enriched liquid formulas (1040 kJ/day) led to a bodyweight increase from the baseline in both groups [127]. Interestingly, diets supplemented with S-MLCT induced a lower increase in body fat mass compared to the LCT supplemented diets (+10% for S-MLCT *vs*. +30% for LCT) [127]. The general metabolic profiles between the two groups were not different, with the exception of glycemia (S-MLCT = 924 *vs*. LCT = 853 mg/100 mL) [127]. A clear link was concurrently established between the propensity of MCT to increase thermogenesis and their previously reported capacity to reduce diet-induced adiposity and body weight-gain [107,128]. Wistar rats submitted to a single oral gavage of 1 g MCT or LCT showed significant elevation in oxygen consumption concomitantly with elevated thermogenesis (1.5 kcal/6 h for MCT *vs*. 0.8 kcal/6 h for LCT) [107]. Another study showed that feeding rats with a diet containing 10% MCT during 6 weeks diminished body fat mass by decreasing subcutaneous and intra-abdominal adiposity compared to a diet containing 10% LCT, suggesting that dietary-MCT lowered body fat mass by inducing thermogenesis [107]. Interestingly, MCT also lower caloric intake, compared to LCT, by improving satiety [129,130]. Octanoate, which constitutes most of the MCT used in dietary interventions, was suggested to be the main co-substrate of the Ghrelin *O*-acetyltransferase enzyme, involved in the activation of the powerful orexigenic peptide hormone Ghrelin [131,132,133]. However, an acute diminution of food intake induced by the consumption of MCT oil is associated with an elevation of Leptin, a hormone with a well-known anti-orexigenic effect [129]. These unsolved discrepancies between different studies highlights the necessity to better understand the molecular effects of MCT and their physiologic implications [132].

Consistent observations related to anti-obesity effects of MCT were generated through animal and human clinical studies. Daily consumption (for 12 weeks at breakfast) of bread supplemented with 14 g S-MLCT (produced by a transesterification of MCT and LCT) led, in comparison with control LCT, to a mild but significant decrease in body weight, and a decrease in subcutaneous and visceral adiposity as well as in total cholesterol level. However, the other metabolic and anthropometric parameters did not significantly vary [134]. The impact of MCT consumption on body-weight is noticeably variable depending on studies and diet protocol designs. Women on a one-month diet where 30% of total energy was supplied by MCT displayed improved fat oxidation and energy expenditure compared to women on LCT-containing meals, and showed a tendency towards decreasing body weight [135]. Interestingly, studies suggested that the catabolic effects induced by the dietary MCT may depend on BMI. In humans, MCT consumption for 4 weeks induced energy expenditure, fat oxidation and body weight loss inversely proportional to BMI [136]. A recent observation has been also made on the impact of MCFA diets on cardiac functions [137]. Compared to LCFA, a 2-week administration of a 38% fat diet containing mostly MCFA positively altered plasma lipids and potentially improved cardiac function as well as insulinemia in adult type 2 diabetics. However, this diet did not significantly impact cardiac TG load or cardiac steatosis [137].

Nevertheless, the consumption of MCT is systematically associated with higher energy expenditure compared to LCT. Solid evidences support the feasibility of using MCT preparations for nutritional trials against obesity, considering their sources, their flexible use and their safety. Compared to soybean oil (providing high amounts of LCT), coconut oil, which is the most easily obtained source of MCT, improves (in synergy with exercise) the cardiometabolic and anthropometric profiles (including waist circumference) of women presenting unhealthy abdominal obesity [138]. However, coconut oil contains other compounds such as antioxidant polyphenols also known to improve metabolic profile by lowering LDL and VLDL while increasing HDL [138,139]. St-Onge and collaborators demonstrated that consumption of a combination of MCT, phytosterols and omega-3-enriched flaxseed oil for 1 month compared to olive oil or beef tallow-based diets, led to improvement in plasma lipid profile in both healthy and overweight women as well as in MHO men [140,141]. In addition, MCT oils can be incorporated into a weight loss program without any adverse effects on metabolic health [142]. Table 1 summarizes the main studies that have revealed the antiobesity potential of MCT.

Consistent with these observations, a recent meta-analysis of randomized controlled trials, combining a broad spectrum of publication resources, showed that MCT administered for 3 weeks or more reduced body weight (−0.51 kg average), waist circumference (−1.46 cm average), hip circumference (−0.79 cm average), total body fat, total subcutaneous fat and visceral fat in comparison with LCT and despite high heterogeneity [143]. Such observations strongly support the relevance of using MCT to modulate body weight and metabolic profile in MUHO patients.

#### 4.2.3. MCT-Supplemented Diets Improve Gut Microbiota and Intestinal Health

MCT are known for their antimicrobial properties. MCT, as well as their constituent MCFA, when provided by maternal milk (along with endogenous long chain unsaturated monoglycerides), exerted antimicrobial effects on the gastro-intestinal tract of suckling neonates and contributed to reduce pathogen transmission [145,146,147]. Moreover, MCT and MCFA were also shown to reduce proliferation of certain species of *Malassezia*, an infectious fungus widespread in hospitals [148].

Kono and collaborators demonstrated that MCT could prevent LPS-mediated endotoxemia. Rats were fed MCT or corn oil (a source of LCT) by daily gavage for 1 week, prior to an intravenously acute dose of endotoxic LPS. Interestingly, while LPS injection led to mortality in corn oil-fed animals, this mortality was prevented in MCT-fed rats [149]. Furthermore, in contrast with the corn oil/LPS group, the MCT/LPS group showed a lower liver injury and inflammation as revealed by a significant decrease in CD14 and tumor necrosis factor alpha (TNF-α) expression in Kupffer cells. In parallel with this, while the corn oil/LPS combination induced a significant increase of intestinal permeability compared to corn oil without LPS, the MCT/LPS combination was associated with a significant improvement of intestinal permeability [149]. In addition, MCT gavage prevented intestinal atrophy (a current problem occurring during parenteral nutrition) and massively reduced (10-fold) the bacterial fecal content [149]. This study showed that MCT administration prevented CD14-activation dependent endotoxemia mediated by LPS, a model consensually associated to obesity and metabolic syndrome.

Most studies on juvenile models use piglets because of their similarities with humans during intestinal development [150,151,152,153]. MCT supplements can be used as alternatives to antibiotics against certain types of porcine colitis [152,154,155]. MCT-fed piglets had a better gastro-intestinal health, with improved intestinal apoptotic index and mucosal turnover, and lowered intraepithelial lymphocyte infiltration (reflecting reduced local inflammation and diminished immune response) compared to controls [152,153]. Furthermore, piglets fed with MCT supplements display a marked modulation of microbial gastric and intestinal populations [152,153,156]. This contributed to a decrease in intestinal inflammation and to an improvement of gut health and integrity [157] with a direct impact on the gut bacteria from Gram-positive (low LPS) and Gram-negative (high LPS) subdivisions [156]. Another study showed that dietary organic acids (OA) and MCFA changed the gut microbiota of weaning piglets [151]. MCFA were shown to reduce the pH of the digesta because of diminished bacterial acid production. OA and MCFA altered the population distribution of *Bacteroidetes*/*Porphyromonas*/*Prevotella* phyla and *Clostridia*/*Streptococcus* genus in a tissue specific manner (stomach, jejunum, ileum and colon). MCFA specifically modulated the bacterial populations in specific regions such as jejunum and colon by promoting the growth of *Escherichia*/*Hafnia*/*Shigella* bacteria and *Clostridia* genus [151]. Combining SCFA and MCFA may constitute a potent tool for the management of optimal gut microbiota. This combination as well as other sources of MCT including coconuts oils, functional mixed oils, or purified MCT oils may be therefore useful for remodelling MUHO gut microbiota and improving metabolically unhealthy obesity. Table 2 summarizes these advances.

## 5. Synthesis and Conclusions

**Figure 1:**
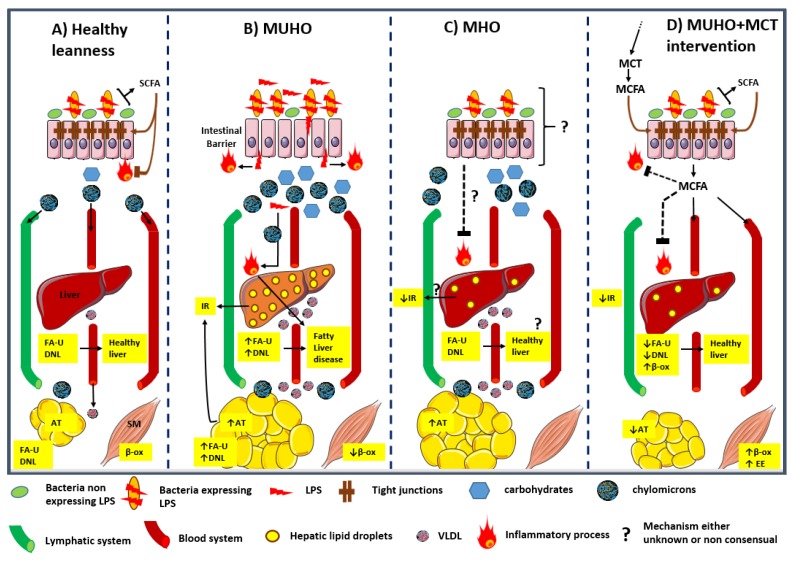
Crosstalk between gut, liver and peripheral metabolic tissues under 4 metabolic states. Under condition of healthy leanness (**A**) an optimal relative abundance of LPS-expressing *vs*. non-expressing bacteria contribute to gut impermeability, low intestinal and hepatic inflammation, and non-obesogenic/steatogenic nutrient supply. Under MUHO conditions (**B**), an elevation in the relative abundance of LPS-expressing bacteria (Gram-negative) induces LPS infiltration and leads to altered intestinal barrier integrity, local inflammation, liver injury and endotoxemia. At the same time, a high fat and carbohydrate supply contributes to adiposity, hepatic steatosis and peripheral insulin resistance. In MHO subjects (**C**), despite an adiposity sustained by a rich diet, a balanced gut microbiota would contribute to maintain intestinal and systemic metabolic health, prevent endotoxemia, and lower hepatic injury and peripheral insulin resistance. Our hypothetical model (**D**) suggests that diet MCT supplementation for MUHO subjects may facilitate a shift towards an MHO-like profile by improving lipid catabolism and lowering adiposity in part, but also by remodelling the gut microbiota into a metabolically beneficial structure. SCFA: short chain fatty acids; FA-U: Fatty acid uptake; AT: adipose tissue; DNL: *de novo* lipogenesis; SM: skeletal muscle; MUHO: metabolically unhealthy obese (or obesity); IR: Insulin resistance; β-ox: beta-oxidation; MHO: metabolically healthy obese (or obesity); MCT: medium chain triglycerides; MCFA: medium chain fatty acids; LPS: lipopolysaccharides; VLDL: very low density lipoproteins.

This review aimed to highlight several aspects underlying the condition of obese subjects, which can be either metabolically unhealthy obese (MUHO) or metabolically healthy obese (MHO). While a panel of criteria serves to define the MUHO state, those defining metabolically healthy obesity remain the subject of current discussions. We underline the necessity of better defining the potential role of gut microbiota in the establishment of MUHO or MHO states. Moreover, we believe that gut microbiota structure may not only serve as a biomarker of those metabolic states, but can also be subjected to a diet-induced remodelling, modifying in turn the metabolic status of patients (MUHO *vs*. MHO or lean state). Dietary MCT, taken alone or with other supplements (such as prebiotics, probiotics, organic acids, *etc*.) could be used as anti-obesity interventions, in regards to their capacity to prevent intestinal permeability/endotoxemia by remodeling gut microbiota, and to prevent unhealthy storage by improving the lipid catabolism/anabolism balance. Figure 1 illustrates this concept.

## Figures and Tables

**Table 1:** Antiobesity effects of MCT and MCFA.

Model	Main Reported Effects for MCT or MCFA	References
Hepatocyte	Downregulated expression of genes involved in DNL and fatty acid uptake; promoted lipid catabolism; reduced steatosis; prevented deleterious lipid accumulation	[108,113,115]
Rat	Lowered TG accumulation in the liver; reduced alcoholic steatosis	[116]
Rat	Decreased body weight gain and body fat mass; lowered fat accumulation and visceral adiposity; did not affect protein assimilation nor metabolism	[107,117]
Rat	Resulted in a higher induction of oxygen consumption and thermogenesis	[107]
Human	Significantly increased postprandial oxygen consumption, energy expenditure, and fat oxidation, in a MCT dose-dependent manner and at a greater extend for lower BMIs	[119,120,121,122,135,136,142,144]
Human	Decreased global adiposity, body fat, and whole-body subcutaneous adipose tissue loss, waist circumference; significantly lowered rate of variation of body fat percentage	[127,130,134]
Human	Did not improve global adiposity	[135]
Human	Did not elevate postprandial circulating TG; did not modulate glucose response, insulinemia and circulating TG levels; lowered LDL/HDL ratio, total and HDL-cholesterol; improved cardiometabolic profile	[129,134,136,138,141]
Human	Promoted rise in leptin and peptide YY	[129]

MCT: medium chain triglyceride; MCFA: medium chain fatty acid; DNL: *de novo* lipogenesis; TG: triglyceride; BMI: body mass index; LDL: low density lipoprotein; HDL: high density lipoprotein; Peptide YY: peptide tyrosine tyrosine.

**Table 2:** Antimicrobial and gut-managing effects of MCT.

Model	Main Reported Effects	References
*Malassezia*	Supressed growth of *M. sympodialis* and *M. furfur*	[148]
Rats	Prevented acute LPS administration-induced mortality, liver injury, liver inflammation, gut impermeability and injury; blunted LPS-induced endotoxemia	[149]
Rats	Significantly blunted TNBS-induced colitis; improved both colonic MPO activity and colonocytes-expressed inflammatory markers	[155]
Rats	Improved gut integrity; modulated immune response to LPS; improved intestinal secretion of IgA	[158]
Piglets	Lowered intestinal pH, in synergy with OA; modulated several gut microbial taxa, potentially preventing postweaning diarrhea	[151]

*M. sympodialis*: *Malassezia sympodialis*; *M. furfur*: *Malassezia furfur*; LPS: lipopolysaccharide; TNBS: 2,4,6-trinitrobenzene sulphonic acid; MPO: myeloperoxidase.
